# The disrupted molecular circadian clock of monocytes and macrophages in allergic inflammation

**DOI:** 10.3389/fimmu.2024.1408772

**Published:** 2024-05-28

**Authors:** Julia Teppan, Juliana Schwanzer, Sonja Rittchen, Thomas Bärnthaler, Jörg Lindemann, Barsha Nayak, Bernhard Reiter, Petra Luschnig, Aitak Farzi, Akos Heinemann, Eva Sturm

**Affiliations:** ^1^ Otto Loewi Research Center for Vascular Biology, Immunology and Inflammation, Division of Pharmacology, Medical University of Graz, Graz, Austria; ^2^ Otto Loewi Research Center for Vascular Biology, Immunology and Inflammation, Division of Immunology, Medical University of Graz, Graz, Austria; ^3^ Ludwig Boltzmann Institute for Lung Vascular Research, Graz, Austria; ^4^ Department of Surgery, Division of Thoracic and Hyperbaric Surgery, Medical University of Graz, Graz, Austria; ^5^ Otto Loewi Research Center, Division of Physiology, Medical University of Graz, Graz, Austria

**Keywords:** molecular circadian clock, macrophages, inflammation, circadian proteins, REV ERB agonist SR9009, inverse ROR agonist SR1001

## Abstract

**Introduction:**

Macrophage dysfunction is a common feature of inflammatory disorders such as asthma, which is characterized by a strong circadian rhythm.

**Methods and results:**

We monitored the protein expression pattern of the molecular circadian clock in human peripheral blood monocytes from healthy, allergic, and asthmatic donors during a whole day. Monocytes cultured of these donors allowed us to examine circadian protein expression in human monocyte-derived macrophages, M1- and M2- polarized macrophages. In monocytes, particularly from allergic asthmatics, the oscillating expression of circadian proteins CLOCK, BMAL, REV ERBs, and RORs was significantly altered. Similar changes in BMAL1 were observed in polarized macrophages from allergic donors and in tissue-resident macrophages from activated precision cut lung slices. We confirmed clock modulating, anti-inflammatory, and lung-protective properties of the inverse ROR agonist SR1001 by reduced secretion of macrophage inflammatory protein and increase in phagocytosis. Using a house dust mite model, we verified the therapeutic effect of SR1001 *in vivo*.

**Discussion:**

Overall, our data suggest an interaction between the molecular circadian clock and monocytes/macrophages effector function in inflammatory lung diseases. The use of SR1001 leads to inflammatory resolution *in vitro* and *in vivo* and represents a promising clock-based therapeutic approach for chronic pulmonary diseases such as asthma.

## 1 Introduction

Circadian rhythms are patterns of a 24-h cycle that involve recurring physical, mental, or behavioral changes. In mammals, the suprachiasmatic nucleus of the hypothalamus functions as a central pacemaker. Responses of this central clock to environmental cues such as light, stress, or food intake are conveyed into the periphery. Thereby, the central pacemaker coordinates autonomous, peripheral circadian clocks, which are present in almost every organ and cell of the body. On a molecular level, the peripheral circadian clocks are driven by interacting transcriptional/translational feedback loops, which are connected by a pair of transcription factors, the Circadian locomotor output cycles kaput (CLOCK): brain and muscle Arnt-like protein-1 (BMAL1) heterodimer. In this study, we focus on the so-called accessory loop of the molecular circadian clock, which includes the repressing nuclear receptor family REV ERB and the activating related orphan receptors (RORs). REV ERB and ROR proteins compete for binding sites within the BMAL1 promoter, with ROR initiating BMAL1 transcription and REV ERB inhibiting it. As target genes overlap, the balance between the two nuclear receptor families is crucial for a dynamic circadian rhythm (1, 2).

There is clear evidence that disruption and disturbances within this circadian system promote inflammation, especially in the respiratory tract, which can progress to inflammatory diseases such as allergic rhinitis (3), asthma (4, 5), or COPD (6). Bidirectional crosstalk between immune cells and the molecular circadian clock emphasizes its essential role in the development of various inflammatory disorders (7). For instance, shift workers have more sleep disruption and an increased risk of cardiovascular disease and asthma (4). Castanon-Cervantes and colleagues demonstrated using a murine chronic jet lag model that constant disruption of the circadian system by shifted dark/light cycle triggered increased proinflammatory cytokine release by innate immune cells and systematic inflammatory diseases (8). Furthermore, the diurnal fluctuations of inflammatory cytokines in response to toxins are abolished in mice lacking Bmal1 or are suppressed by pharmacological manipulation of the circadian proteins (9, 10).

Nuclear receptors are ligand-activated transcription factors that represent one of the largest and most commonly targeted families of druggable proteins (11). However, modulating the molecular circadian clock can result in both anti- and proinflammatory consequences. Nowadays, there are various synthetic ligands of nuclear circadian receptors that have been tested in experimental settings (12, 13). For instance, the REV ERBα agonist SR9009 significantly suppressed LPS-induced inflammatory responses of bone-marrow-derived macrophages (BMDMs) from Bmal1 knockout mice (14). Furthermore, Sitaula et al. showed that SR9009 reduced the polarization of BMDMs to classical activated M1 macrophages while promoting the polarization to M2 macrophages (15). In recent studies, also the inverse ROR agonist SR1001 has been shown to possess anti-inflammatory properties (16–18) and specifically modulate macrophage function. Treatment with SR1001 reduces the viability of macrophages derived from a human monocytic leukemia THP-1 cell line (19).

In general, macrophages play an essential role in pulmonary immune homeostasis and are a pivotal source of proinflammatory cytokines and chemokines under pathological conditions (20). It is known that macrophages can either originate from peripheral monocytes or develop from an embryonic precursor before birth such as tissue-resident lung macrophages (21). If needed, circulating monocytes migrate into the lungs, where they differentiate into monocyte-derived alveolar macrophages (Mo-AMs) and regulate a proinflammatory response (22). Depending on environmental signals, macrophages polarize to different phenotypes, in a simplified model either to classically lipopolysaccharide (LPS) and interferon gamma (IFN-γ)-activated macrophages involved in tissue damage (so-called M1 macrophages) or to an alternatively interleukin (IL)-4/IL-13-activated subtype, contributing to wound healing and the resolution of inflammation (so-called M2 macrophages) (23, 24). Thus, an inflammatory stimulus in the lungs can also lead to phenotype switching of tissue-resident alveolar or interstitial lung macrophages from M2 dominant to M1 cells. Thus, macrophage dysfunction, including phenotype switching or altered phagocytic behavior, is closely associated with respiratory diseases, especially asthma pathology, where both M1- and M2-like phenotypes are found (25).

Asthma is known as a heterogenous chronic inflammatory lung disease with a strong circadian signature. For instance, symptoms and diagnostic markers show diurnal variations (26–28). In this study, we therefore wanted to further investigate the role of the molecular circadian clock in monocytes and macrophages, particularly during inflammation, and to clarify whether clock-modulating ligands impact macrophage effector function. Our results suggest that the molecular circadian clock is active in human monocytes and macrophages and responsive to inflammatory mediators. Furthermore, clock-targeting ligands such as the inverse ROR agonist SR1001 may be of therapeutic interest, as they restore the balance between a pro-inflammatory and an anti-inflammatory macrophage phenotype.

## 2 Materials and methods

### 2.1 Ethical approval

All experiments involving primary cells from human subjects were approved by the Institutional Review Board of the Medical University of Graz (EK 17–291 ex 05/06). All volunteers signed an informed consent. Blood donors were divided into healthy, non-allergic, and allergic donors due to serological test. Information on asthma diagnosis and medication was self-reported by the participants. The collection of human lung samples was approved by the Institutional Ethics Board (32–446 ex 19/20), following the patients’ informed consent. All studies involving animal experiments were approved by the Animal Ethics Committee of the Austrian Federal Ministry of Science and Research and carried out in line with the European Community’s Council Directive (2022–0.626.093).

### 2.2 Whole blood staining

For whole blood samples, leukocyte populations were stained with CD3-APC-Cy7 (Biolegend, California, USA, 317342), CD14-BV421 (Biolegend, 301830), and CD16-PerCP-Cy5.5 (Biolegend, 301828). Cells were fixed with FIX (Nordic-MUbio, Netherlands, GAS-002) and stored in staining buffer (PBS + 2%FBS) overnight at 4°C. On the next day, all samples were contemporaneously permeabilized using PERM (Nordic-MUbio, Netherlands, GAS-002). Samples were then blocked with human TruStain FcX™ Fc Receptor Blocking Solution (Biolegend, 422302), and circadian proteins were stained intracellularly using the following primary antibodies: NR1D1 (Abcam, United Kingdom, ab174309), REV ERB beta (Novusbio, Colorado, USA, NBP2–19576), BMAL1 (Novusbio, NB100–2288), CLOCK (Mybiosource, California, USA, MBS4750976), ROR alpha (Thermo Fisher Scientific, Massachusetts, USA, PA1–812), ROR beta (Novusbio, NBP1–82532), and ROR gamma-PE (R&D Systems, Minnesota, USA, IC6006P). The secondary donkey anti rabbit-PE antibody (Biolegend, 406421) was used for detection. Samples were measured on a BD FACSCanto II flow cytometer and analyzed as FI-FMO using FlowJo 10.8.1. Afterwards, Z-scores were calculated and normalized to TP 0 of the healthy control.

### 2.3 Generation of human monocyte-derived macrophages

Human peripheral blood mononuclear cells (PBMCs) were isolated from citrated blood by density gradient sedimentation. Peripheral blood monocytes in the PBMC fraction were resuspended in pre-warmed adhesion medium (RPMI supplemented with 5% human AB serum, non-essential amino acids, sodium pyruvate, HEPES, and 1% penicillin/streptomycin) at a concentration of 10 Mio cells/mL and seeded onto Corning^®^ CellBIND plates (Sigma-Aldrich, Missouri, USA) for 2 h at 37°C in a humidified atmosphere with 5% CO_2_. Non-adherent cells were washed off and enriched monocytes incubated with differentiation medium [10% FCS, 1% penicillin/streptomycin, and 20 ng/mL recombinant human (rh) M-CSF (Peprotech, New Jersey, USA, AF-300)] for a week. Cultured human monocyte-derived macrophages (MDMs) were polarized with 20 ng/mL rh IFN-γ (Immunotools, Germany, 11343534) and 100 ng/mL LPS (Sigma-Aldrich, L2880) or 20 ng/mL rh IL-4 (Immunotools, 11340043). To ensure a good viability, a live dead staining was performed using zombie green (FITC-labeled, Biolegend, 42311, 1:500 dilution). Polarization was confirmed in accutase-detached macrophages by staining with monoclonal antibodies CD80-BV421 (Biolegend, 305222, 1:100 dilution) and CD206-APC-Cy7 (Biolegend, 321120, 1:100 dilution) for 30 min.

### 2.4 Agonist treatment

During polarization, human MDMs were treated with REV ERB or ROR agonists. Based on literature research, a concentration of 10 µM SR9009 (29), 10 µM SR1001 (15), or 10 µM SR1078 (30) was used for *in vitro* experiments.

### 2.5 Intracellular staining

Cells were fixed and permeabilized with FIX and PERM (Nordic-MUbio, GAS-002). Thereafter, the samples were blocked with human TruStain FcX™ Fc Receptor Blocking Solution (Biolegend, 422302), and circadian proteins were stained intracellular using primary antibodies NR1D1 (Abcam, ab174309), BMAL1 (Novusbio, NB100–2288), and ROR beta (Novusbio, NBP1–82532). The secondary donkey anti rabbit-PE (Biolegend, 406421) antibody was used for detection. Samples were measured by flow cytometry and analyzed as an increase over the isotype control.

### 2.6 Precision-cut lung slices (PCLS)

PCLS were obtained as described by Rittchen et al. (31). The collection of human lung samples during tumor resection was approved by the Institutional Ethics Board (32–446 ex 19/20), following the informed consent of the patients. The fresh lung tissue was obtained during tumor resections. To generate PCLS, only the non-tumorous tissue was used. In brief, cylindrical cores (8 mm in diameter) were sliced (sections of 200 μm ± 20 μm) using a Krumdieck live tissue microtome in Earle’s balanced salt solution containing 25 mmol HEPES and 17 mmol glucose. Media were changed to incubation medium containing sodium-pyruvate, MEM amino acids, MEM vitamins, and L-glutamine (all Thermo Fisher Scientific) and penicillin/streptomycin, and the tissue was incubated in a humidified atmosphere with 5% CO_2_ at 37°C. The incubation medium was changed at least five times after 45-min wash periods. On the following day the media were changed again followed by the stimulations: PCLS were either left untreated or activated with 100 ng/mL LPS and 20 ng/mL IFN-γ or 20 ng/mL rh IL-4 and 20 ng/mL rh IL-13. Conditioned media and tissue were collected 6 h or 24 h after activation. Tissue was fixed with formalin for 1 h at room temperature and embedded in paraffin.

### 2.7 Immunofluorescent staining

Serial cut PCLS (4 µm) were deparaffinized, followed by a heat-induced antigen retrieval in sodium citrate buffer (pH 6). Unspecific binding was blocked with 4% BSA and 10% goat serum in PBS for 2 h at room temperature. Primary antibodies BMAL1 (Novusbio, NB100–2288, 1:100 dilution) and CD68 (Abcam, ab955, 1:100 dilution) were diluted in 1:10 diluted blocking solution, and slides were incubated overnight at 4°C. After washing steps, sections were incubated with secondary antibodies Cy3-labeld donkey anti-rabbit and goat anti-mouse conjugated with Alexa Fluor 488 (both from Thermo Fisher Scientific, 1:500) for 2 h. Nuclear counterstaining was performed with the DAPI containing mounting medium TrueVIEW^®^ Autofluorescence Quenching Kit (Vector Laboratories, California, USA). Images were taken with a fluorescence microscope.

All images were acquired using constant laser settings with an Olympus IX73 fluorescence microscope. Controls included unstained cells, slides stained with either BMAL1 only or CD68 only, and secondary antibodies only. Images were automatically analyzed with the same threshold settings using Image J. In brief, FITC-channel CD68+ positive cells were considered as macrophages, and the fluorescence intensity of BMAL1 was measured in Cy3 channel within these cells.

### 2.8 Cytokine multiplex assay

The supernatant from macrophages was used to measure the concentrations of granulocyte colony stimulating factor (G-CSF), interleukin 8 (IL-8), monocyte chemoattractant protein 1 (MCP-1), monokine induced by interferon gamma (MIG), and macrophage inflammatory protein (MIP) 1α and 1β using the human chemokine 6-plex FlowCytomix from eBioscience (California, USA) according to the manufacturer’s instructions.

### 2.9 Phagocytosis

A Vybrant Phagocytosis assay (Thermo Fisher Scientific, V-6694) was performed and analyzed according to the manufacturer’s recommendation and measured by flow cytometry. In brief, fluorescein-labeled *Escherichia coli* (K-12 strain) was internalized by the treated macrophages, followed by trypan blue staining to quench the fluorescence from the non-absorbed particles.

### 2.10 Clock gene expression

Microarray data from Woodruff et al. (32) were analyzed to explore clock gene expression in alveolar macrophages comparing 15 non-smoking healthy controls and 15 non-smoking asthmatic patients. The data discussed in this publication were accessed through GEO Series accession number GSE2125 (https://www.ncbi.nlm.nih.gov/sites/GDSbrowser?acc=GDS1269#details).

### 2.11 Animal experiment

To develop a mixed phenotypic model of allergic airway inflammation, the application route, doses, and timing of sensitization was adjusted from Tan et al. representing different asthma phenotypes in mice using house dust mite (HDM), which is among common allergens inducing asthma (33, 34). Herein, 12-week-old BALB/c mice were challenged intranasally with 10 µg of HDM allergen (dissolved Acarizax SLIT-tablet in PBS) four times once a week to induce an HDM allergy model. Dissolved SLIT tablets that did not contain any allergen were used as a control. Afterwards, the automated home cage phenotyping LabMaster system (TSE Systems, Germany) was employed to analyze possible effects of SR1001 on the circadian pattern in singly housed mice. As recommended by the company and supported by several studies, SR1001 was injected i.p. a single dosage of 25 mg/kg (35–37). The locomotion, exploratory behavior, and water and food intake of the test mice (age: 8–12 weeks) were continuously recorded in the home-like environment of the LabMaster system (TSE Systems, Bad Homburg, Germany) as previously described (38). For this purpose, transparent LabMaster experimental cages were surrounded by two frames emitting infrared beams to measure vertical exploratory behavior and horizontal locomotor activity by counting infrared beam interruptions. In addition, two weight sensors attached to the cage lids were used to assess ingestive behavior as a food container and a drinking bottle were attached to the sensors throughout the experiment. All recording devices were connected to a personal computer, which was used to record and analyze the data using LabMaster software. Food and water intake were recorded in grams of food (g) and milliliters of water (mL), respectively. Before starting the agonist treatment, the animals were habituated to the food containers, and the drinking bottles used in the LabMaster system and to single housing for 3 days. In the LabMaster system, the mice need to be housed individually to allow accurate activity measurements. Animals were placed into LabMaster cages to habituate the mice to single housing and the used drinking bottles. After a 3-day habituation period, mice were treated with SR1001 [25 mg/kg/twice a day (=bd)] i.p. or with vehicle every 12 h, in total for five times. All LabMaster parameters were recorded during the treatment period until bronchoalveolar lavage fluid (BALF) was collected under anesthesia. Immune cell populations were separated by flow cytometry using CD11b-PE-Cy7, CD11c-BV421, Ly6G-APC, and Siglec-F-PE antibodies to gate for alveolar macrophages.

### 2.12 H/E staining

Mice lung sections of 5 μm were deparaffinized and stained with hematoxylin and eosin (H/E) staining and observed using a Olympus IX73 fluorescence microscope. Six to eight photomicrographs at ×100 magnification were taken and automatically evaluated in a blind fashion by ImageJ.

### 2.13 Statistical analyses

All data are shown as mean ± SEM for n observations. Correlations were created and analyzed with RStudio (PBC, Boston, MA, URL http://www.rstudio.com/). Other statistical analyses were performed using GraphPad Prism software 6.0 (La Jolla, CA, USA). To identify statistical outliers, ROUT or Grubbs test was performed. After Kolmogorow–Smirnow test, groups were compared by t-test or Mann-Whitney-U-Test, one‐ or two‐way ANOVA followed by respective *post-hoc* test. Probability values of p < 0.05 were considered statistically significant and are indicated as *p < 0.05, **p < 0.01, and ***p < 0.005.

## 3 Results

### 3.1 The molecular circadian clock of peripheral monocytes and alveolar macrophages is disrupted in asthmatic patients

**Figure 1:**
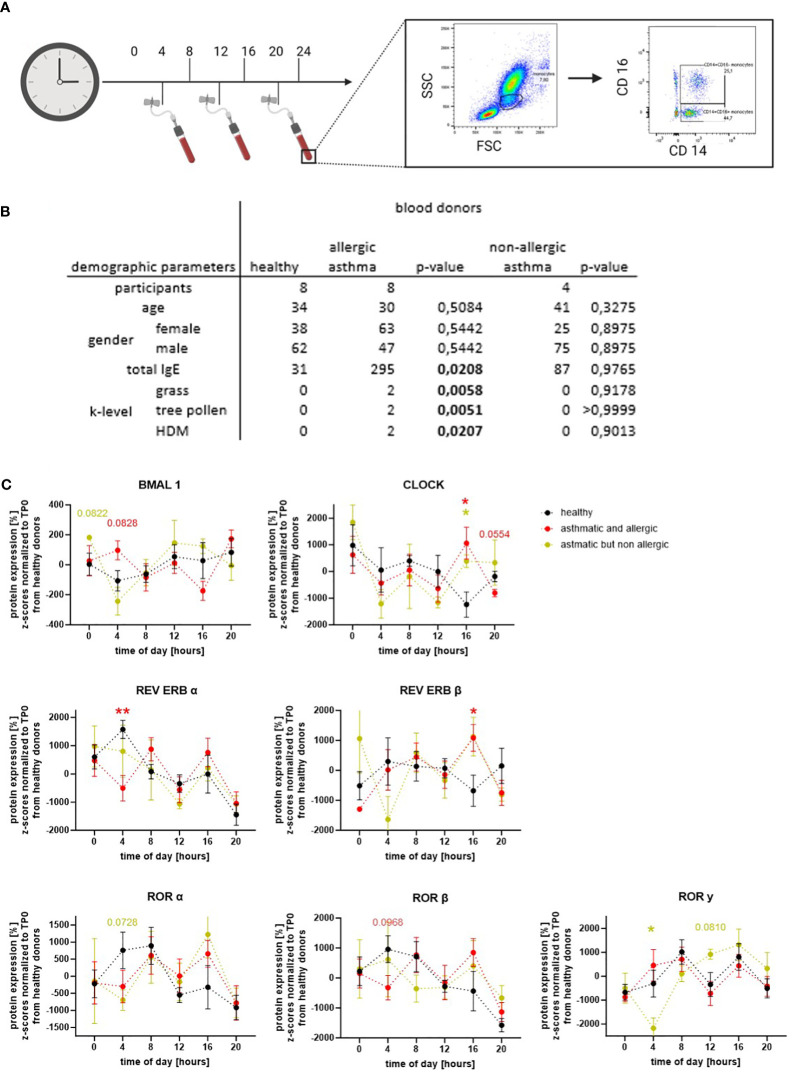
Circadian proteins have an oscillating expression pattern in human classical monocytes. **(A)** Blood was drawn three times per day and partly incubated to detect the expression level of circadian proteins every 4 h. CD14+CD16− monocytes were gated according to size and marker staining. **(B)** Demographic table summarizing three blood donor groups: healthy donors (black), asthmatic/allergic donors (red), and non-allergic asthmatic (ocher) based on their self-reported asthma diagnoses from an external respiratory physician and serological tests. **(C)** Oscillating expression pattern of BMAL1, CLOCK, REV ERBs, and RORs were detected in CD14+CD16− monocytes (n ≥ 4). For statistical analyses, Z-scores were calculated and normalized to the mean of the healthy control group. Expression levels between the blood donor groups were compared within one time point. Mean ± SEM: ROUT outlier test, Kolmogorow–Smirnow test, two-way ANOVA, and Dunnett’s *post-hoc* test. *p = 0,05, **p < 0.01.

To confirm that the expression of circadian proteins in human peripheral monocytes is oscillating in a daytime specific manner, a whole blood staining protocol was established. To this end, blood was collected from healthy, allergic, and/or asthmatic volunteers three times a day (4 a.m., 12 p.m., and 8 p.m.). One part of the blood was stained for flow cytometry immediately, while the second part was incubated at 37°C and processed 4 h later. In this way, a total of six time points a day could be recorded. For flow cytometric analysis, cells were divided by FSC/SSC and surface markers to gate for classical CD14+CD16− monocytes (**Figure 1A**). The abundance of intermediate and non-classical monocytes was too low to distinguish those two populations; however, analyses of CD14+CD16+ cells are shown in **Supplementary Figure S1**. Based on self-reported allergic symptoms and asthma diagnosed by external respiratory physicians, and total and specific IgE values, participants were assigned to the following three groups: healthy donors, allergic asthmatic donors, and non-allergic asthmatic donors (**Figure 1B**).

Our results clearly show that the circadian proteins of the accessory loop (BMAL1, CLOCK, REV ERBs, and RORs) are expressed in classical peripheral monocytes of healthy, allergic, and/or asthmatic donors. Furthermore, oscillating protein patterns during the course of the day were found for all detected circadian proteins in the three groups (**Figure 1C**). In monocytes from healthy donors, the core protein BMAL1 showed a low overall amplitude but increased expression towards the evening. For CLOCK, the binding partner of BMAL1, peak protein expression was observed at midnight, and lowest levels were found at 4 p.m. As expected, the repressor of BMAL1, REV ERBα, showed a protein expression pattern opposite to BMAL1: highest levels were detected at 4 a.m., while lowest protein expression was found at 8 p.m. The second member of this nuclear receptor family, REV ERBβ, showed a reduced overall oscillation amplitude with nadirs at 4 p.m. and midnight. Interestingly, a higher protein expression of the BMAL1 activators RORα and β was observed during the first half of the day, while RORγ oscillation showed a shorter phase but higher amplitudes peaking at 8 a.m. and 4 p.m. As illustrated in **Figure 1C**, the oscillation of the circadian proteins of the accessory loop, with the exception of CLOCK, had shorter phases, but higher amplitudes in allergic asthmatics. In addition, when comparing protein expression at specific time points, a significant decrease in REV ERBα and a trend towards increased BMAL1 at 4 a.m., a time closely associated with severe asthma attacks and nocturnal asthma, was observed. Tendencies towards lower expression levels were also seen in the activating receptor family ROR at this time point. However, a maximum in the protein expression of RORα and β, but also of REV ERBβ and CLOCK, was observed at 4 p.m. The oscillation of RORγ did not differ between healthy and allergic asthmatic donors. Interestingly, in healthy donors, the expression pattern of RORγ differed from RORα and β, while in allergic asthmatics, there was more similarity. The only clear difference between healthy donors and the small group of non-allergic asthmatics was a significant drop in RORγ and a trend towards reduced RORα at 4 a.m. These results clearly indicate that the molecular circadian clock of classical monocytes is altered or even disrupted in asthma, depending on the atopy status of the patients.

**Figure 2:**
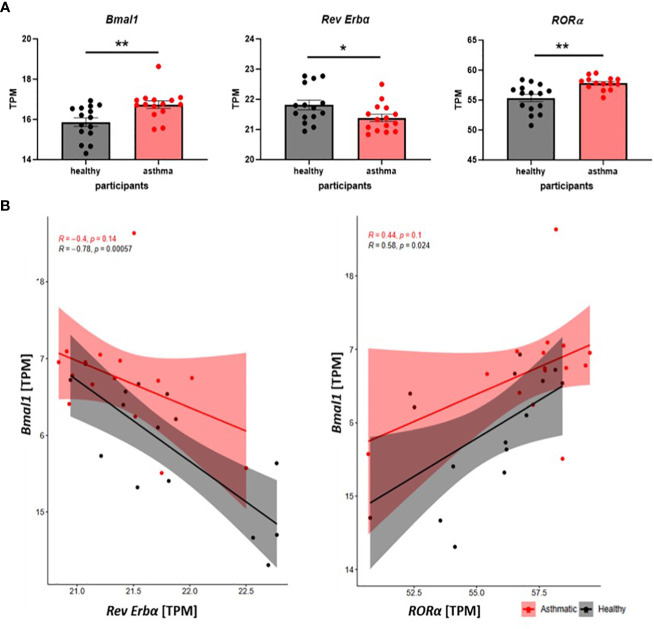
The molecular circadian clock is disrupted in alveolar macrophages of asthma patients. **(A)** Clock gene expression in alveolar macrophages of healthy and asthma patients is compared on mRNA level (transcripts per kilobase million =TPM) using microarray data, which are accessible through GEO series (GSE2125). Mean ± SEM; Grubbs outlier test, Kolmogorow–Smirnow test, t-test, and Mann–Whitney. **(B)** Scatter plots of correlation show the interplay of *Bmal1* and *Rev Erbα* and *Bmal1* with RORα in AMs from healthy (black) and asthmatic (red) participants. Pearson correlation, *p < 0.05, **p < 0.01.

Next, we took advantage of a publicly available data set using microarray data published by Woodruff and colleagues (32) to compare clock gene expression in alveolar macrophages (AMs) of healthy donors and asthmatic patients. Similar to our observations from patient-derived monocytes, significant differences in the clock protein expression were observed. BMAL1 and its activator RORα were significantly increased, while, accordingly, the mRNA levels for the repressor Rev Erbα were reduced in AM from asthmatics (**Figure 2A**). Furthermore, the well-described positive correlation of BMAL1 and RORα and the negative feedback loop of Rev Erbα repressing the main orchestrator Bmal1 was lost in asthma-derived AMs (**Figure 2B**). These data further emphasize the circadian disruption and the associated loss of balance of the peripheral clock in alveolar macrophages during chronic inflammatory diseases such as asthma.

### 3.2 The protein expression of the accessory loop in human macrophages depends on the inflammatory environment

#### 3.2.1 Altered circadian protein expression upon *in vitro* polarization in macrophages from allergic individuals

**Figure 3:**
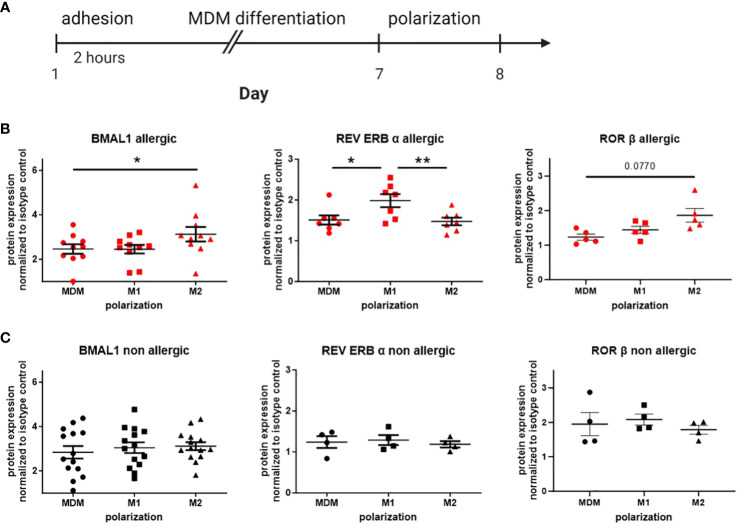
Circadian protein levels in macrophage subtypes differ from allergic and healthy blood donors. **(A)** Human peripheral monocytes were seeded in 12-well plates and differentiated into monocyte-derived macrophages (MDMs). Afterward, MDMs were further polarized to M1 or M2 with LPS/IFN-γ or IL-4, respectively. Expression levels of the circadian proteins BMAL1, REV ERBα, and RORβ in MDM, M1, and M2 were detected by flow cytometry in cultures from **(B)** allergic (n ≥ 5) and **(C)** healthy blood donors (n ≥ 4). Fold increase over isotype control (IC) was calculated. Mean ± SEM; ROUT outlier test, one-way ANOVA, *p < 0.05, **p < 0.01.

In peripheral blood monocytes, main differences in circadian protein expression were found between healthy donors and patients with allergic asthma. Thus, in the following experiment, we explored the protein expression of the accessory loop in monocyte-derived macrophages (MDMs) from healthy and allergic blood donors at a single time point. First, isolated PBMCs were cultured and differentiated to MDMs for 7 days and further polarized with LPS/IFN-γ (M1) or IL-4 (M2) (**Figure 3A**). Dividing daily blood donors in allergic and healthy participants, altered circadian protein expression in response to macrophage polarization was only observed in allergic donors, but not in the healthy group (**Figures 3B, C**). In cells derived from allergic donors, macrophage polarization with IL-4 leading to a M2 phenotype resulted in significantly increased BMAL1 protein expression compared to unpolarized MDMs. Furthermore, REV ERBα, the repressor of BMAL1, was found to be significantly lower in M2 compared to LPS/IFN-γ-polarized M1 macrophages. The core clock protein BMAL1 and its activator RORβ were significantly increased in IL-4-polarized cells from allergic donors compared to unpolarized macrophages, while the main repressor REV ERBα was significantly increased in the M1 phenotype (**Figure 3B**).

Given the pronounced differences in circadian protein levels among macrophage subtypes derived from allergic blood donors, our subsequent aim was to directly compare unpolarized and polarized macrophages from allergic and non-allergic donors. M1-polarized macrophages from healthy and allergic blood donors revealed significant alterations in circadian protein expression, characterized by an increase in REV ERBα expression and correspondingly lower levels of RORβ. Additionally, these cells displayed reduced BMAL1 expression compared to M1 macrophages from non-allergic individuals (**Supplementary Figure S2B**). Interestingly, a similar trend in RORβ was also observed in unpolarized macrophages (**Supplementary Figure S2A**), while REV ERBα was only slightly increased in M2 macrophages from allergic donors (**Supplementary Figure S2C**).

These results suggest that the molecular circadian clock of macrophages from allergic patients is disrupted and thus responds differently to polarization into both proinflammatory and resolution-associated phenotypes.

#### 3.2.2 BMAL1 protein expression of human lung-resident macrophages depends on the inflammatory stimulus

**Figure 4:**
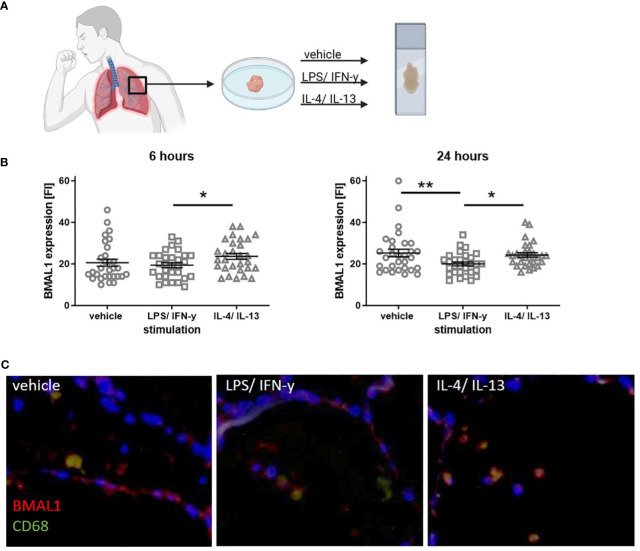
Stimulation with LPS/IFN-γ or IL-4/IL-13 leads to an altered BMAL1 expression in human lung-resident macrophages. **(A)** Serial cut PCLS from a human lung tissue from non-tumor resections were cultured and stimulated with LPS/IFN-γ or IL-4/IL-13 for either 6 or 24 h. **(B)** Co-staining of the macrophage marker CD68 and BMAL1 showed that resident macrophages express BMAL1. Six hours of IL-4/IL-13 stimulation increased BMAL1 expression compared to LPS/IFN-γ treatment. Similarly, after 24 h of IL-4/IL-13 stimulation, BMAL1 expression was more pronounced, whereas 24 h of LPS/IFN-γ resulted in significantly lower BMAL1 levels. Fluorescence intensity **(FI)** was calculated with ImageJ. **(C)** Representative images of 24-h stimulated PCS are shown (scale bar, 50 μm). CD68 is shown in green, and BMAL1 is labeled in red. Mean ± SEM; ROUT outlier test, t-test, *p < 0.05, **p < 0.01.

Here, we used human precision cut lung slices (PCLS) as an *ex vivo* approach to further confirm that BMAL1 protein expression reflects the inflammatory state of the lung tissue. Therefore, PCLS from human lung tissue from non-tumor resections were stimulated with either LPS/IFN-γ or IL-4/IL-13 for 6 or 24 h. Immunofluorescence microscopy was used to detect BMAL1 protein expression (**Figure 4A**). Similar to our previous *in vitro* results from allergic donors, higher BMAL1 levels were detected in lung-resident macrophages from IL-4/IL-13-treated compared to LPS/IFN-γ-treated PCLS after 6 and 24 h. A 24-h stimulation even resulted in a significantly lower BMAL1 expression in LPS/IFN-γ-treated compared to vehicle-treated lung slices (**Figures 4B, C**). Thus, it appears that in tissue-resident lung macrophages, BMAL1 is regulated in opposite directions depending on the inflammatory milieu and phenotype of the cells.

### 3.3 The polarization state and effector function of macrophages is altered by clock-modulating ligands targeting REV ERB or ROR

#### 3.3.1 The synthetic REV ERB agonist SR9009 favors inflammatory behavior of human MDMs

Since we observed disease-associated changes in the protein expression of BMAL1 and its regulatory proteins REV ERB and ROR, we aimed to investigate the potential therapeutic effects of synthetic clock-modulating ligands. To explore the proposed anti-inflammatory properties of the REV ERBα agonist SR9009 on macrophage polarization, MDMs were again cultured and activated to M1 and M2 macrophages. Afterwards the expression of macrophage surface markers CD80 and CD206, which display M1 or M2 activation, respectively, was evaluated to prove successful polarization.

**Figure 5:**
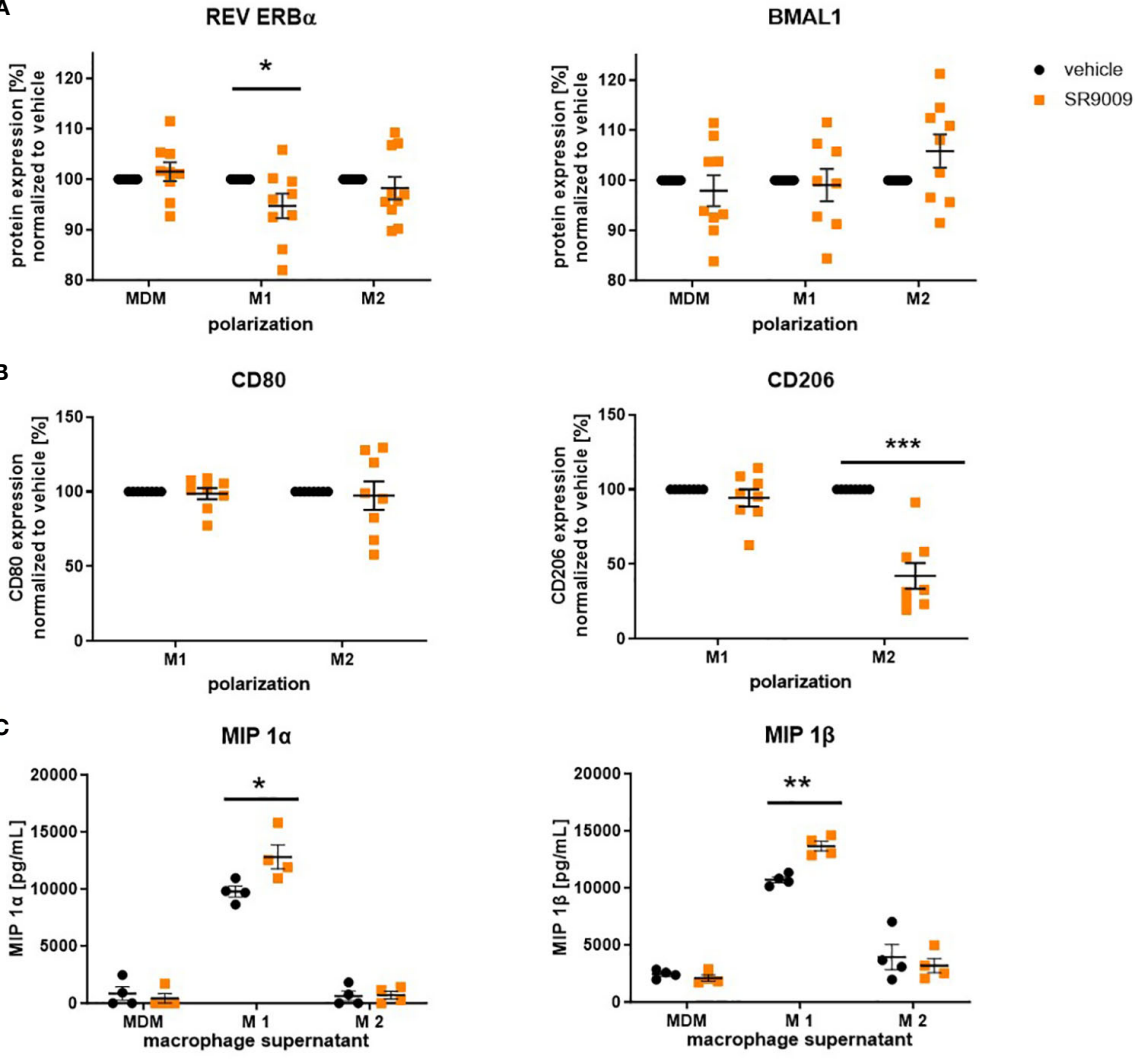
The REV ERBα agonist SR9009 induces a proinflammatory phenotype switch in human macrophages. Macrophages were differentiated from human peripheral PBMCs and polarized with LPS/IFN-γ or IL-4 in the presence or absence of SR9009 (10 µM). **(A)** Expression levels of the circadian protein REV ERBα and BMAL1 were detected by intracellular flow cytometric staining (n ≥ 8. **(B)** Macrophages were pretreated with REV ERB antagonist SR8278 5 h before polarizing them in the presence of the agonist SR9009. Polarization marker CD80 and CD206 were detected by flow cytometric surface staining (n ≥ 8). **(C)** Higher concentrations of macrophage inflammatory proteins (MIPs) were measured in the supernatant of SR9009-treated M1 macrophages using a human chemokine 6-plex assay (n ≥ 4). Mean ± SEM; Grubbs outlier test, t-test, or one-way ANOVA, *p < 0.05, **p < 0.01, ***p < 0.001.

As illustrated in **Figure 5A**, SR9009 decreased REV ERBα expression in M1 and slightly increased BMAL1 in M2 macrophages, which, on the one hand, confirms its clock-modulating potential but, on the other hand, also indicates rather proinflammatory effects of this synthetic agonist. Interestingly, we also observed a decreased surface expression in CD206, pointing to a reduced shift towards the M2 phenotype. However, consistent with previous REV ERBα-independent observations by Dierickx and colleagues (39), pre-treatment with the REV ERB antagonist SR8278 neither blocked nor reversed the effect of SR9009 on M2 polarization (**Figure 5B**).

Moreover, increased concentrations of the proinflammatory macrophage inflammatory proteins MIP 1α and MIP 1β, which are strongly associated with allergic airway inflammation (40, 41), were detected in supernatants of SR9009-treated M1 macrophages (**Figure 5C**).

#### 3.3.2 The inverse ROR agonist SR1001 shows anti-inflammatory properties *in vitro* and *in vivo*

Due to the unfavorable proinflammatory properties and the evident REV ERBα-independent effects of SR9009, we subsequently targeted the opponent of REV ERB, the ROR receptor family, by using the inverse ROR agonist SR1001.

**Figure 6:**
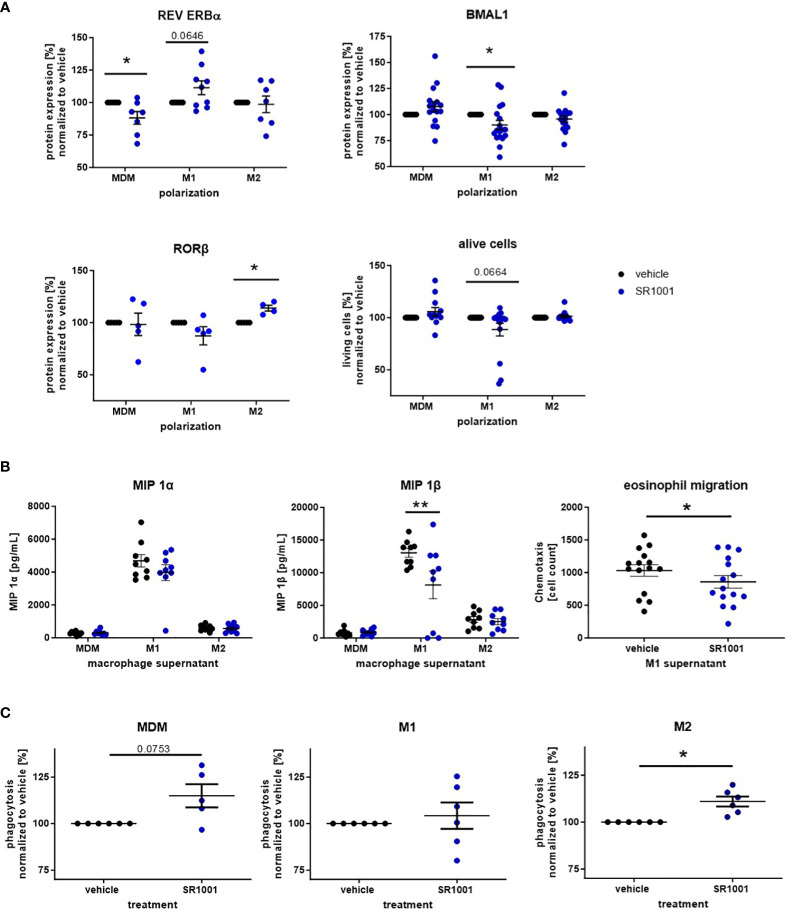
The inverse ROR agonist SR1001 exhibits anti-inflammatory properties in human monocyte-derived macrophages. Macrophages were differentiated from human peripheral PBMCs and polarized with LPS/IFN-γ or IL-4. Additionally, cells were treated with SR1001 (10 μM) during polarization. **(A)** The expression levels of the circadian proteins BMAL1 and REV ERBα were detected by intracellular flow cytometric staining (n ≥ 5); cell viability was monitored using a zombie dye (n ≥ 10). **(B)** Concentrations of macrophage inflammatory proteins (MIPs) were measured in supernatants using a human chemokine 6-plex assay (n ≥ 8). Eosinophil migration assays were performed with a microBoyden chemotaxis chamber (n=15). **(C)** Phagocytosis of human MDM and polarized macrophages was detected with the Vybrant Phagocytosis kit and analyzed according to manufacturer’s instruction (n ≥ 5). Mean ± SEM; Grubbs outlier test, t-test, or one-way ANOVA, *p < 0.05, **p < 0.01.

As expected, SR1001 treatment also influenced circadian protein expression in MDMs and polarized macrophages. In more detail, the expression of the BMAL1 repressor REV ERBα was reduced in MDMs in response to SR1001 treatment but was found more pronounced in M1-polarized macrophages (**Figure 6A**). As a consequence, SR1001-treated M1 macrophages showed significantly lower BMAL1 levels, which was accompanied by a tendency towards reduced viability of the cells (**Figure 6A**). In M2 macrophages, only RORβ levels significantly increased.

As opposed to SR9009, treatment with SR1001 decreased the secretion of MIP, especially MIP 1β, by M1 macrophages (**Figure 6B**). Both MIP 1α and MIP 1β are chemokines for eosinophil granulocytes and stimulate the recruitment of eosinophils into the asthmatic airways (42, 43). Hence, we explored the migratory responsiveness of purified human eosinophils towards the supernatants from M1-polarized macrophages. Indeed, significantly fewer eosinophils migrated towards the supernatant of SR1001-treated macrophages compared to the vehicle group (**Figure 6B**).

Several studies reported impaired phagocytosis by airway and monocyte-derived macrophages from asthmatic patients (44–46). Moreover, it has been shown that the phagocytic activity of macrophages oscillates during the day in a BMAL1-dependent manner (47). Hence, we also explored the phagocytic potential of SR1001-treated macrophages *in vitro* and observed increased phagocytic capacity of MDMs and M2 macrophages (**Figure 6C**), whereas the ROR agonist SR1078 significantly decreased the phagocytic potential in M1 macrophages (**Supplementary Figure S3**).

These results highlight the anti-inflammatory properties of the inverse ROR agonist SR1001 that inhibited proinflammatory chemokine release from M1 macrophages and augmented the phagocytic activity from M2-like cells.

**Figure 7:**
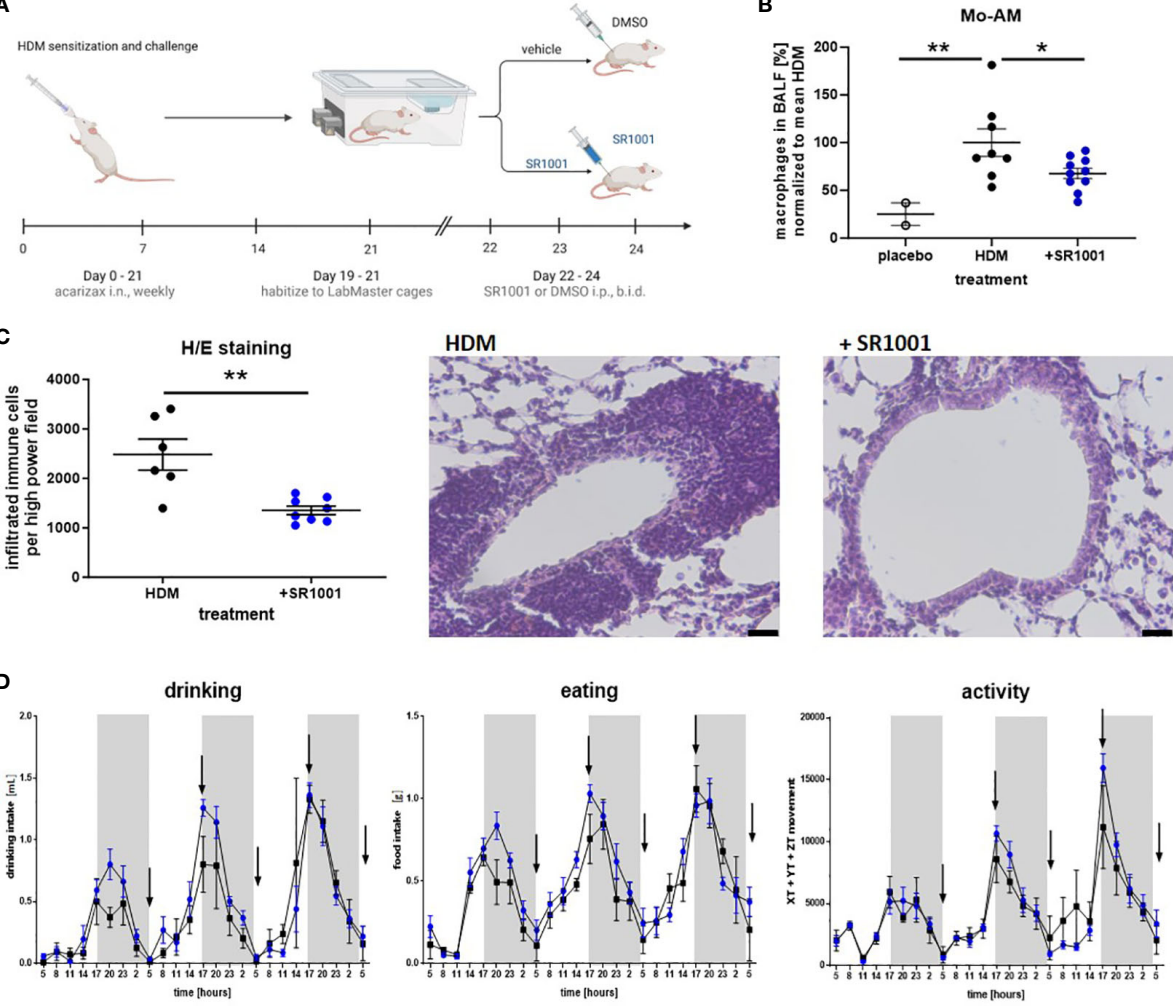
The inverse ROR agonist SR1001 shows anti-inflammatory properties *in vivo*. **(A)** HDM model: mice were treated i.n. with dissolved SLIT tablets (10 μg), which contain 12 SQ-HDM standardized allergen extract from HDM, once a week for 4 weeks. Dissolved SLIT tablets without allergen were used as a control. On day 19, mice were placed into the LabMaster cages for 3 days to habituate. Starting from day 22, mice were treated five times i.p. with SR1001 (25 mg/kg) or DMSO as a vehicle twice a day (b.i.d.), indicated by black arrows. After the last SR1001 injection on day 24, BALF was collected. Schematic figure created with BioRender.com. **(B)** Immune cells derived from BALF were evaluated for CD11c^high^ Siglec-F^low^ monocyte-derived alveolar macrophages (Mo-AM) by flow cytometry (n ≥ 8). **(C)** H/E staining was performed and automatically analyzed using ImageJ. Representative images are shown (scale bar, 10 μM, n ≥ 6). **(D)** Circadian home-cage behavior (drinking, eating rhythm, and physical activity) of mice was recorded every minute by the automated home cage phenotyping LabMaster system (n ≥ 5). Mean ± SEM; ROUT test, t-test, two-way ANOVA, *p < 0.05, **p < 0.01.

In addition to the observed anti-inflammatory potential of the inverse ROR agonist SR1001 *in vitro*, we further confirmed the *in vivo* relevance of SR1001 as a therapeutic approach in a murine model of house dust mite (HDM)-induced lung inflammation, which mimics key features of human asthma such as allergen-driven inflammation and eosinophilic infiltration (48). Therefore, following intranasal HDM challenge, mice were injected five times every 12 h with SR1001 or vehicle control (**Figure 7A**). As presented in **Figure 7B**, the treatment significantly reduces the proportion of proinflammatory Mo-AMs in the BAL fluid of mice, which corresponded to our *in vitro* data showing a trend towards reduced M1 survival in human macrophages (**Figure 6A**). Furthermore, this reduction in Mo-AMs was associated with a significant decrease in immune cell infiltration into the lung tissue and reduced goblet cell hyperplasia (**Figure 7C**). As RORα has been associated with the regulation of circadian behavior (49), the behavior of the mice (drinking behavior, eating rhythm, and physical activity) was constantly monitored during the treatment by using the LabMaster system. Importantly, no significant differences were observed comparing treated animals and control group, despite inverse RORα agonist treatment (**Figure 7D**). Therefore, these results demonstrate the anti-inflammatory and lung-protective properties of SR1001 without affecting the general circadian rhythm.

## 4 Discussion

In the present study, we used different *in vitro* and *in vivo* approaches to show that a functionally active but imbalanced molecular circadian clock is present in human peripheral blood monocytes and in monocyte-derived and tissue-resident lung macrophages under allergic and/or asthmatic conditions. The protein levels of the components of the accessory loop seem to reflect the prevailing inflammatory milieu as observed in monocytes and monocyte-derived macrophages from allergic patients, macrophage polarization experiments, and in an experimental model of HDM-induced airway inflammation. The disease-associated disruption of the circadian clock in monocytes and macrophages seems to drive forward the chronic inflammation, and thus, clock-modulating ligands might represent a novel therapeutic approach by supporting an anti-inflammatory macrophage phenotype without affecting daily biorhythm.

Although recent studies have demonstrated that clock genes oscillate in many peripheral tissues such as the lung (50–52), their properties in the human immune system are still quite unknown. In addition, most circadian research on monocytes and macrophages has so far produced contradictory results, as it has only been carried out at the mRNA level or in mouse macrophages. Boivin et al. were among the first to demonstrate oscillating expression of the central clock loop in human PBMCs (53). Hence, we first wanted to investigate the oscillating pattern of the molecular circadian clock on a protein level in human peripheral blood monocytes. For this purpose, we chose a similar study design as Baumann et al., who demonstrated that the molecular clock of peripheral blood cells remains active for several hours after blood collection, when incubated at 37°C (54). Indeed, we are the first to report significant differences in the amplitude and phase of the respective clock proteins between asthmatic and healthy subjects depending on the time of day and the atopy status of the patients. It should be emphasized that a significant reduction in the anti-inflammatory repressor REV ERBα and a related increase in BMAL1 at 4 a.m., a time strongly associated with severe asthma attacks, was only observed in allergic asthmatics but not in non-allergic asthmatic patients. Furthermore, the significant increase in the second repressor REV ERBβ at 4 p.m. was accordingly reflected by a drop in BMAL1. At the same time, the BMAL1 binding partner CLOCK was significantly increased in allergic asthmatics, which is in line with a previous finding from Kondratov et al. indicating that the highest levels of CLOCK are detected in cells with low BMAL1 levels (55). Interestingly, the BMAL1 activator and Th17-lineage-specific transcription factor RORγ was unaltered in allergic asthmatics but showed a significantly different protein oscillation in terms of amplitude and phase in non-allergic asthmatics. Thus, this monitoring experiment supports our hypothesis that circadian proteins from monocytes are altered under inflammatory conditions and also reflect the specific inflammatory environment. However, further studies in a larger cohort and additional monitoring of the core loop are required to better understand the regulation of the molecular circadian clock in monocytes under distinct inflammatory conditions.

Analysis of AM derived from asthmatics and healthy controls revealed differences in the clock gene expression including Bmal1, Rev Erbα, and RORα. A significant increase in the mRNA expression level of Bmal1 and RORα was found during asthma. Accordingly, Rev Erbα was found to be significantly decreased compared to AMs from healthy controls. More importantly, these changes led to a loss of balance within the molecular circadian clock in asthmatics as reflected by a lack of correlation between the activator RORα and Bmal1 and the repressor Rev Erbα and Bmal1 (56).

It is well-known that macrophages polarize into different phenotype subsets depending on the inflammatory milieu and that this process is altered in allergic and asthmatic patients. Based on the observation that the most pronounced differences in monocyte circadian proteins were observed in patients with allergic asthma, we differentiated human peripheral blood monocytes to MDMs and LPS/IFN-γ or IL-4-polarized cells and compared the protein expression levels of the accessory loop between non-allergic and allergic donors. Several *in vitro* studies revealed that macrophages differentiated from peripheral monocytes exhibit circadian oscillation in clock gene expression. Nevertheless, these oscillating patterns experience a decrease in their amplitude during cell culture due to the absence of external environmental stimuli (57). Our results clearly show that the circadian protein expression directly responds to the inflammatory environment, as polarization-induced changes were only observed in cells from allergic donors. BMAL1 and its activator RORβ were significantly increased in IL-4-polarized macrophages, similar to asthma-derived AM macrophages, while interestingly, levels of the BMAL1 repressor REV ERBα were increased in M1 macrophages. The observed increase in RORβ after IL-4 stimulation is consistent with previous findings by Gopinath et al. who showed that higher RORβ levels indicate an anti-inflammatory environment and a shift to a resolution-promoting macrophage phenotype. While RNAseq is a powerful method, RNA expression may not always align with protein abundance. Alveolar macrophages can exhibit a mixed polarization phenotype and are not exclusively derived from peripheral monocytes (58). Consequently, differences may arise between the circadian protein levels from monocyte-derived macrophages and the mRNA expression levels of clock genes from preparations of alveolar macrophages. All in all, the observed changes within the circadian clock are in line with a recent observation from the Farkas lab showing that the amplitude and rhythmicity of the molecular circadian clock in macrophages is differentially affected based on the polarization stimulus. Similarly, M1 polarization in response to LPS or IFN-γ led to a decrease in the BMAL1 amplitude, while M2 polarization using IL-4 resulted in an opposite effect (59). However, to our knowledge, we are the first to show that this strong circadian responsiveness is primarily observed in cells from allergic donors.

Considering that PCLS retain remarkable cellular complexity and recapitulate cellular interactions in the tissue (60), we used this technique as an *ex vivo* approach to determine BMAL1 expression in tissue-resident macrophages in the human lung. Furthermore, organotypic human PCLS are a well-established model for acute and/or allergic pulmonary inflammation. For instance, stimulation of human PCLS with LPS successfully induced the production of proinflammatory mediators and hence is an adequate model to explore the effects of inflammation in the human lung (31, 61, 62). Thus, we stimulated PCLS with similar mediators as used for macrophage polarization *in vitro*. Both approaches, LPS/IFN-γ or IL-4/IL-13 treatment, which favor either M1 or M2 polarization, lead to similar changes in BMAL1 as observed in monocytes and AM from asthmatics and in cultured macrophages from allergic donors. Compared to LPS/IFN-γ treatment, the combination of IL-4/IL-13 increased BMAL1 levels after 6 and 24 h, while BMAL1 was reduced after 24-h incubation with LPS/IFN-γ when compared with vehicle-treated slides. These data are again consistent with our own *in vitro* data and the previous observations from Farkas et al. and, thus, support the hypothesis that the molecular circadian clock directly responds to tissue inflammation and thus reflects disrupted tissue homeostasis.

The strong connection between the immune system and the circadian rhythm is known to be bidirectional. On the one hand, inflammatory stimuli can alter the circadian rhythm and disturb the balance of the circadian clock leading to manifestation and progression of inflammatory diseases such as asthma. On the other hand, the molecular circadian clock is known to time immune responses (63). Hence, clock-based therapeutic strategies, including chronotherapy, where dosing time is optimized for maximum therapeutic outcome, and pharmacological ligands that specifically modulate the molecular clock might be of great potential for future treatments of inflammatory disease. As REV ERBα is known to be the key link between the immune system and the circadian clock (64) and, furthermore, a lack of REV ERBα enhances lung inflammation (65), we first tested the impact of the REV ERBα agonist SR9009 on *in vitro* polarized macrophages. We observed that SR9009 favors the inflammatory M1 phenotype indicated by a counter-regulation of REV ERBα, which was associated with increased MIP 1α and MIP 1β release. Furthermore, the REV ERB antagonist SR8278 could not reverse the SR9009-induced reduction in M2 polarization. This observation is consistent with previously described REV ERBα-independent effects of SR9009 on cell proliferation, metabolism, and gene transcription (39, 66), but remains to be controversial to other studies demonstrating anti-inflammatory properties of SR9009 in mouse bone-marrow-derived macrophages (14, 15).

Due to these uncertain results, we decided to use the inverse ROR agonist SR1001 for further experiments, which inhibits the activators of the core clock protein BMAL1. In previous studies, SR1001 has already been shown to reduce Th2 inflammation by decreased cytokine production in an inflammatory dermatitis model *in vivo* (16–18). Correspondingly, we demonstrated that SR1001 increased the anti-inflammatory REV ERBα in M1 macrophages and reduced the viability and MIP 1α and MIP 1β secretions in these cells. MIPs are strongly associated with airway inflammation, as increased levels have been found in the BALF of patients with allergic asthma (41). Recently, MIP 1β has been identified as a potent driver of eosinophilic airway inflammation in an ovalbumin-induced model of allergen-induced airway inflammation (67). Accordingly, we noted decreased eosinophil migration towards the supernatant from SR1001-treated M1 macrophages. Moreover, MIPs are not only potent chemoattractants for effector cells of asthma such as eosinophils (42, 43) but also for monocytes and macrophages themselves (68–70). In SR1001-treated M2 macrophages we observed a further increase in RORβ levels, which has been linked with a more pronounced anti-inflammatory phenotype (58).

As macrophages contribute to asthma pathology by altering their function, e.g., by increased cytokine production and impaired phagocytosis (25, 71, 72), we therefore investigated the beneficial potential of SR1001 on the phagocytic capacity of human MDMs, M1 and M2 macrophages. We observed that SR1001 supports macrophage-driven host defense by increasing the phagocytotic activity in unpolarized MDMs and M2 macrophages, which is another promising anti-inflammatory property of this inverse ROR agonist (25). This observation is also in line with a study from Kitchen et al. who identified a gain of phagocytic function due to loss of BMAL1 in macrophages (47). Consequently, when present during *in vitro* polarization, SR1001 diminished the proinflammatory function of M1 macrophages while simultaneously reinforcing and fortifying the anti-inflammatory and pro-resolution properties of M2 macrophages.

Based on our *in vitro* findings and the fact that RORα and Bmal1 are increased in AM from asthmatic patients, we tested the *in vivo* effects of SR1001 in an HDM model of allergen-induced airway inflammation that mimics the main characteristics of human asthma. Here, we found a significant decrease in proinflammatory CD11c^high^ Siglec-F^low^ Mo-AMs in the BALF from SR1001-treated mice, which corresponds very well with the reduced chemokine secretion and M1 viability from our *in vitro* experiments. As inflammation becomes established after repeated allergen exposures, monocytes can be recruited to the lungs, differentiate to macrophages, and orchestrate proinflammatory reactions (22). In addition to these proinflammatory Mo-AMs, tissue-resident alveolar macrophages can polarize to a proinflammatory phenotype, loose their suppressive effects, and gain pathogenic functions, i.e., by releasing proinflammatory cytokines and initiating the recruitment of other inflammatory effector cells (23). Correspondingly, the reduced Mo-AMs in the BALF were associated with decreased immune cell infiltration into the lungs and diminished goblet cell hyperplasia. Thus, targeting alveolar macrophages under inflammatory conditions offers new therapeutic opportunities. Since the inverse ROR agonist was injected systemically, an anti-inflammatory effect independent of macrophages involving other immune cell populations cannot be excluded and needs to be investigated further. In addition, Wei et al. have already stated earlier that systemic application of SR1001 reduced immune cell infiltration, goblet cell hyperplasia, and specific IgE serum levels in an ovalbumin-induced model of allergic rhinitis in mice (73).

To ensure that this exogenously applied inverse agonist represents a suitable treatment approach, despite the involvement of RORα in circadian behavior (49), we monitored the rhythmic behavior of the animals during the experiment. Herein, the LabMaster system was used to ensure a constant home-cage recording of the mice (38). Importantly, no differences in terms of activity including locomotion, drinking, or eating behavior were observed, although the molecular circadian clock was targeted. Of course, local delivery of SR1001 directly into the airways would represent an improved therapeutic approach, minimizing potential systemic side effects, especially when used as a long-term treatment.

In conclusion, our study shows for the first time that the molecular circadian clock is expressed on a protein level in human monocytes and macrophages in an oscillating manner and identified distinct alterations in cells from allergic and/or asthmatic donors. Furthermore, the crucial balance and correlation between the components of the accessory loop is lost in AMs from asthmatic patients. However, targeting the nuclear circadian receptor families REV ERB and ROR as a therapeutic intervention impacts both circadian protein expression and macrophage effector function but can lead to a pro- or anti-inflammatory state. Thus, here, we confirm the clock-modulating agonist impact macrophages and their effector cell functions *in vitro* and *in vivo* including their abundance and chemokine secretion without interfering with the physiological circadian rhythm. Therefore, this study identified clock-modulating ligands as a promising novel steroid-free treatment approach for inflammatory diseases and further encourages research to explore the benefits of clock-based therapies.

## Data availability statement

The original contributions presented in the study are included in the article/**Supplementary Material**, further inquiries can be directed to the corresponding author.

## Ethics statement

The studies involving humans were approved by Institutional Review Board of the Medical University of Graz (EK 17–291 ex 05/06). The studies were conducted in accordance with the local legislation and institutional requirements. The participants provided their written informed consent to participate in this study. The collection of human lung samples was approved by the Institutional Ethics Board (EK 32–446 ex 19/20), following the patients’ informed consent. The animal study was approved by Animal Ethics Committee of the Austrian Federal Ministry of Science and Research and carried out in line with the European Community’s Council Directive (2022-0.626.093). The study was conducted in accordance with the local legislation and institutional requirements.

## Author contributions

JT: Conceptualization, Data curation, Formal analysis, Investigation, Methodology, Project administration, Software, Supervision, Validation, Visualization, Writing – original draft, Writing – review & editing. JS: Data curation, Formal analysis, Investigation, Methodology, Writing – review & editing. SR: Data curation, Investigation, Methodology, Validation, Writing – review & editing. TB: Investigation, Methodology, Visualization, Writing – review & editing. JL: Resources, Writing – review & editing. BN: Writing – review & editing. BR: Validation, Writing – review & editing. PL: Data curation, Writing – review & editing. AF: Investigation, Methodology, Writing – review & editing. AH: Investigation, Writing – review & editing. ES: Conceptualization, Formal analysis, Funding acquisition, Methodology, Project administration, Resources, Supervision, Visualization, Writing – original draft.
