# Supplementary figures and images for: The disrupted molecular circadian clock of monocytes and macrophages in allergic inflammation
Source: Front Immunol. 2024 May 28;15:1408772. doi: 10.3389/fimmu.2024.1408772 (PMC11165079; doi:10.3389/fimmu.2024.1408772)

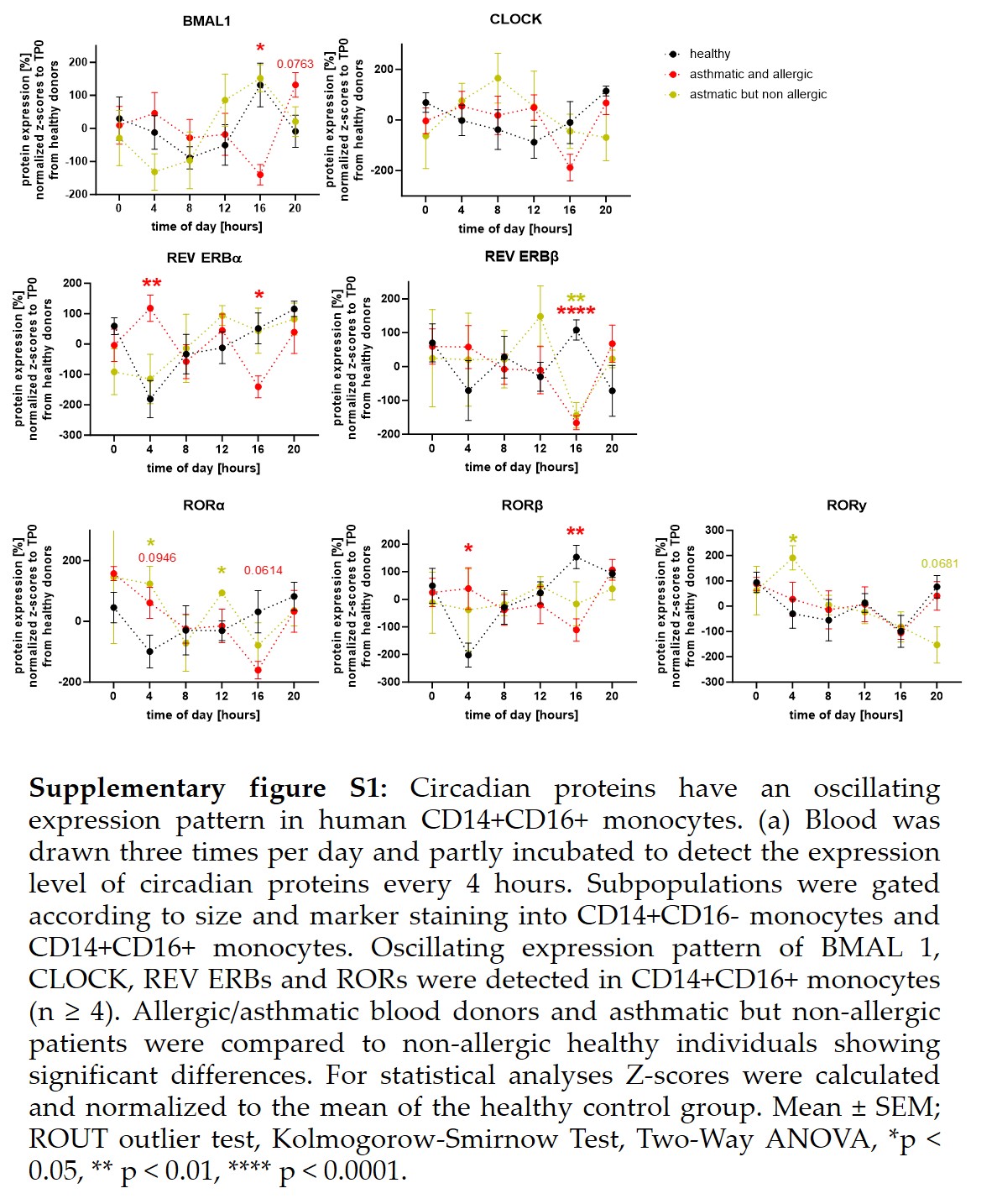

Supplement: Supplementary file 1 [file Image_1.jpg]

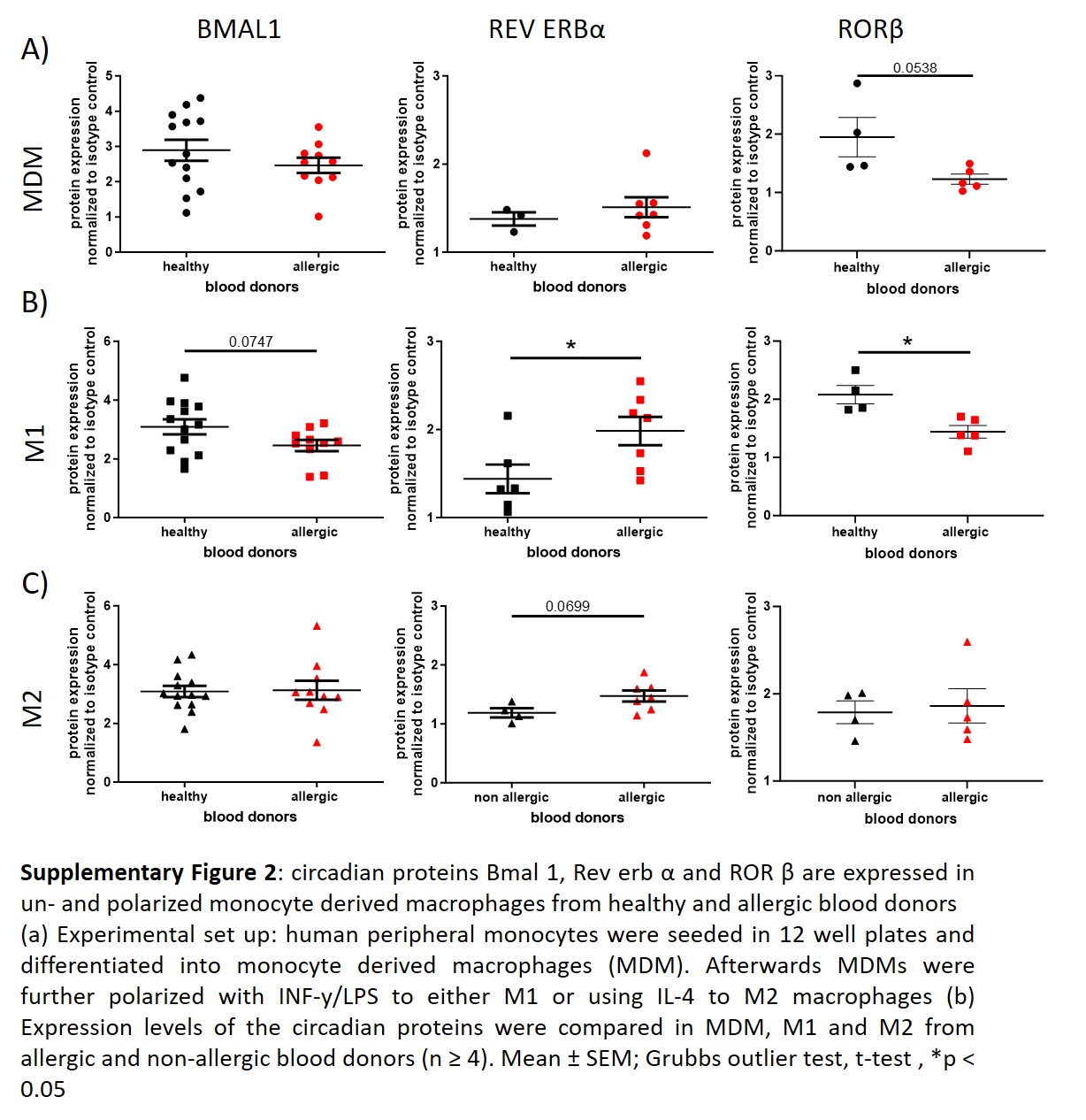

Supplement: Supplementary file 2 [file Image_2.jpg]

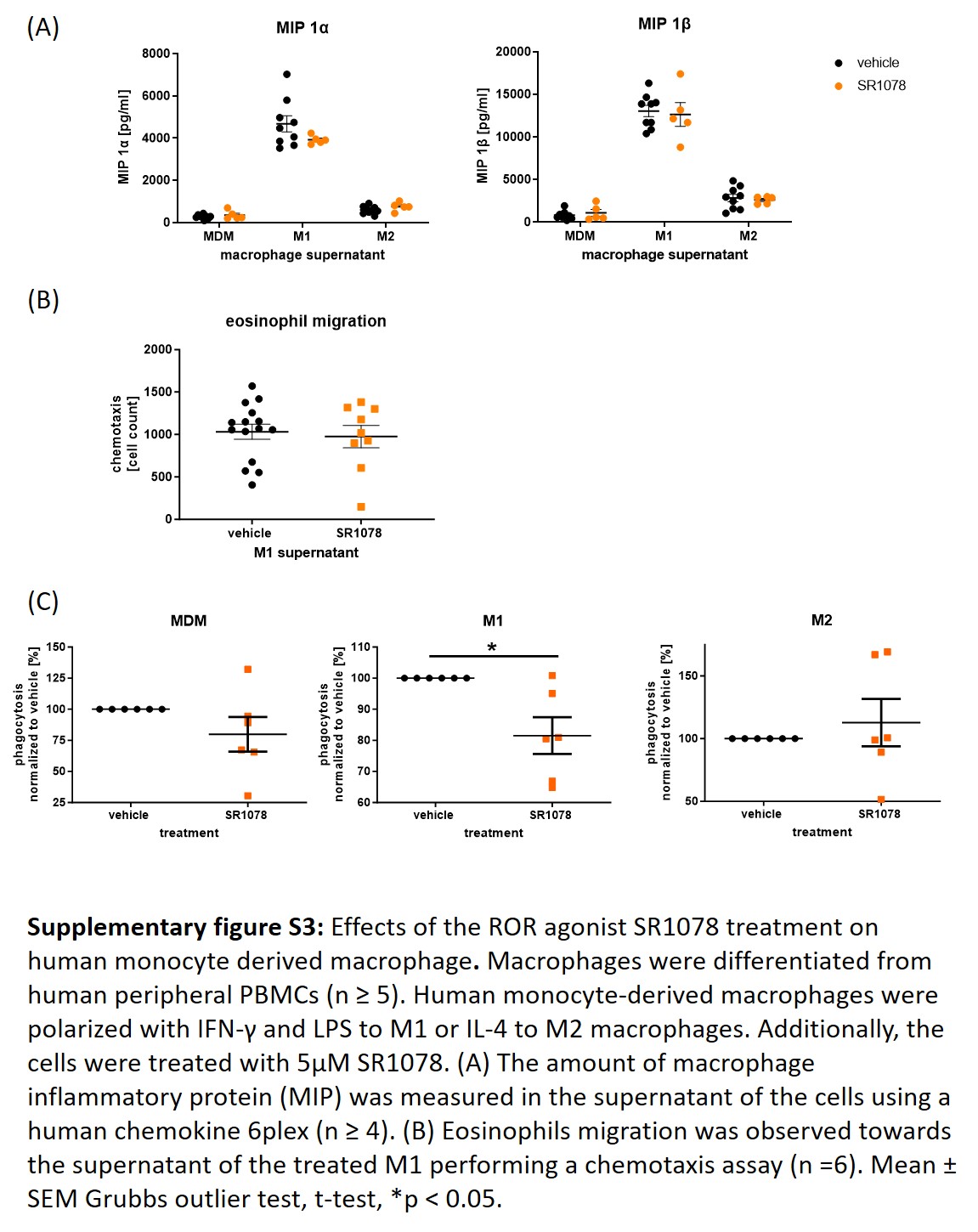

Supplement: Supplementary file 3 [file Image_3.jpg]

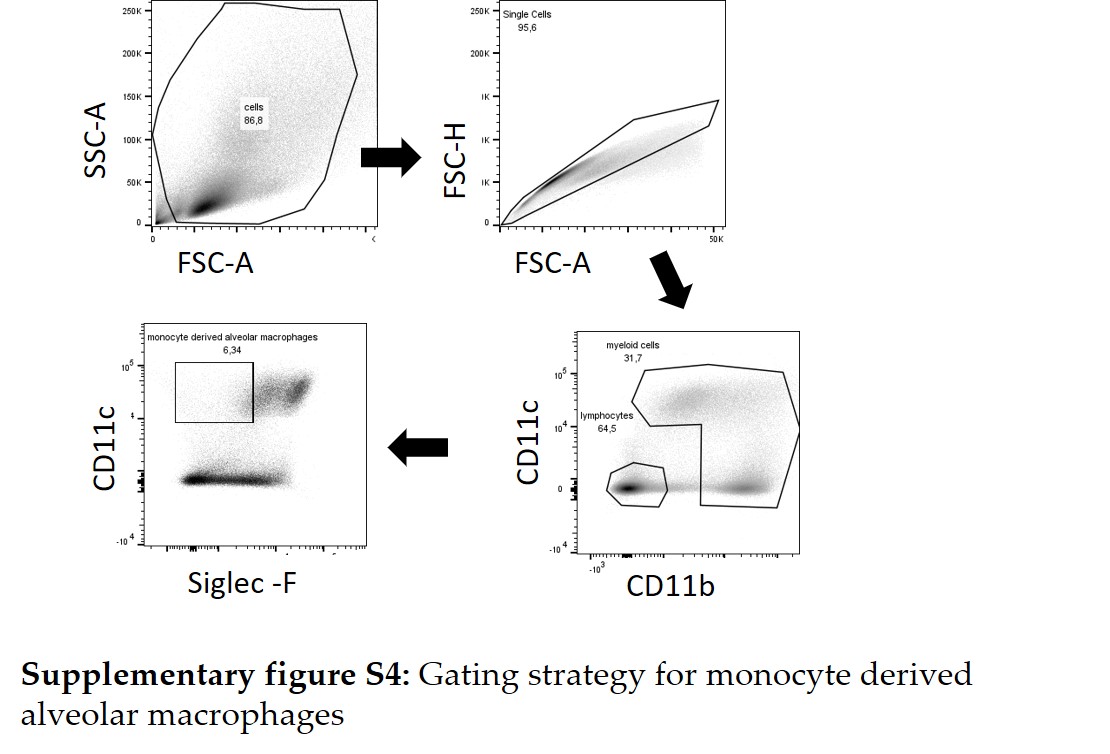

Supplement: Supplementary file 4 [file Image_4.jpg]
